# Microsatellite mapping of QTLs affecting resistance to coccidiosis *(Eimeria tenella*) in a Fayoumi × White Leghorn cross

**DOI:** 10.1186/1471-2164-10-31

**Published:** 2009-01-20

**Authors:** Marie-Hélène Pinard-van der Laan, Bertrand Bed'hom, Jean-Luc Coville, Frédérique Pitel, Katia Feve, Sophie Leroux, Hélène Legros, Aurélie Thomas, David Gourichon, Jean-Michel Repérant, Paul Rault

**Affiliations:** 1 INRA/AgroParisTech, UMR1236 Génétique et Diversité Animales, F-78352 Jouy en Josas Cedex, France; 2 Laboratoire de Génétique Cellulaire, INRA, BP 52627 F-31326 Castanet-Tolosan Cedex, France; 3 LABOGENA, F-78352 Jouy-en-Josas, France; 4 Pôle d'expérimentation avicole de Tours, INRA, F-37380 Nouzilly, France; 5 UR VIPAC, VIPAC – Parasitologie, AFSSA, Zoopôle les Croix, BP 53, F-22440 Ploufragan, France; 6 Syndicat des Sélectionneurs Avicoles et Aquacoles Français, Station de Recherches Avicoles, INRA, F-37380 Nouzilly, France

## Abstract

**Background:**

Avian coccidiosis is a major parasitic disease of poultry, causing severe economical loss to poultry production by affecting growth and feed efficiency of infected birds. Current control strategies using mainly drugs and more recently vaccination are showing drawbacks and alternative strategies are needed. Using genetic resistance that would limit the negative and very costly effects of the disease would be highly relevant. The purpose of this work was to detect for the first time QTL for disease resistance traits to *Eimeria tenella *in chicken by performing a genome scan in an F2 cross issued from a resistant Fayoumi line and a susceptible Leghorn line.

**Results:**

The QTL analysis detected 21 chromosome-wide significant QTL for the different traits related to disease resistance (body weight growth, plasma coloration, hematocrit, rectal temperature and lesion) on 6 chromosomes. Out of these, a genome-wide very significant QTL for body weight growth was found on GGA1, five genome-wide significant QTL for body weight growth, plasma coloration and hematocrit and one for plasma coloration were found on GGA1 and GGA6, respectively. Two genome-wide suggestive QTL for plasma coloration and rectal temperature were found on GGA1 and GGA2, respectively. Other chromosme-wide significant QTL were identified on GGA2, GGA3, GGA6, GGA15 and GGA23. Parent-of-origin effects were found for QTL for body weight growth and plasma coloration on GGA1 and GGA3. Several QTL for different resistance phenotypes were identified as co-localized on the same location.

**Conclusion:**

Using an F2 cross from resistant and susceptible chicken lines proved to be a successful strategy to identify QTL for different resistance traits to *Eimeria tenella*, opening the way for further gene identification and underlying mechanisms and hopefully possibilities for new breeding strategies for resistance to coccidiosis in the chicken. From the QTL regions identified, several candidate genes and relevant pathways linked to innate immune and inflammatory responses were suggested. These results will be combined with functional genomics approaches on the same lines to provide positional candidate genes for resistance loci for coccidiosis. Results suggested also for further analysis, models tackling the complexity of the genetic architecture of these correlated disease resistance traits including potential epistatic effects.

## Background

Coccidiosis is the most important parasitic disease affecting poultry production. There are several species of chicken coccidia, each having a particular host location and characterized by a specific pathogenic effect such as characteristic gross lesions [1]. *Eimeria tenella *is one of the most frequent ones, developing in the caecum, affecting feed conversion, causing depression of body weight gain, lesions and in the most severe cases, mortality. The worldwide cost of coccidiosis to poultry production has been estimated to be over $800 million per year [2]. More interestingly than the approximate global cost, the compartmentalized model for the estimation showed that in the UK, 98.1% of the cost involved broilers and 80.6% were due to the effect of the disease (mortality, weight gain and feed conversion) and 17.5% only to the cost of chemoprophylaxis and therapy [3]. The use of genetic resistance has the potential to limit the negative and costly effects of the disease and hence would be relevant to implement. So far, most commonly used chemotherapeutics are anticoccidial drugs. But the drug resistance phenomena and increasing concerns about an impact on the food chain and environment are limiting their use and alternatives are vaccines with the recent development of research on novel immunoprotective antigens [4,5].

Genetic variability for resistance to coccidiosis in the chicken has been extensively proven to exist by either successful divergent selection for survival to acute infection by *E. tenella *[6], or a large effect of host genetics as measured by comparing mostly inbred chicken lines [e.g. [7,8]] or different chicken pure broiler lines for resistance to *E. acervulina *[9]. Potential effects of the MHC on resistance to *E. tenella *were shown in some congenic lines [e.g. [10]] but not substantiated in other studies including outbred lines [e.g. [11]]. Recently, an effect of MHC on the expression of immune-related cytokine and chemokine genes related to resistance to *E. maxima *was reported [12]. Indeed, to decipher the complexity of the immune mechanisms and their underlying genetic control, immunogenomic approaches [13] are needed in combination with structural genomics approaches.

Using the genetic resistance to coccidiosis would be an attractive alternative control measure. But in the absence of real candidate genes, genetic markers linked to resistance remain to be identified, which could be included in breeding strategies to help poultry breeders to increase genetic resistance to diseases like coccidiosis. The last decade has shown the emergence of new generations of markers which allow such a search [14]. Performing a genome-wide screen of an F2 resource population is one of the possible strategies. To our knowledge, only one genome scan was performed to identify QTL of resistance to coccidiosis, *E. maxima *[15]. One QTL on oocyst production was clearly identified and confirmed on GGA1 [16].

The objective of this present study was to identify QTL for different resistance traits to *E. tenella*. The chosen strategy was to produce, challenge and genotype an F2 cross from two chicken lines, a Fayoumi line and a Leghorn line, identified previously as resistant and susceptible for *E. tenella*, respectively [11]. The analysis of the F2 and results of the genome scan are presented here.

## Results

### Characterization of resistance traits to *E. tenella *in the F0 (Fayoumi and Leghorn), F1 and F2 populations

Phenotypic trait means and standard deviations for lesion (LES), body weight gain (WG) and mortality in each F0 line (Leghorn and Fayoumi), in their F1 and F2 cross, and in the 15% selected susceptible F2 (F2-S) and 15% selected resistant F2 (F2-R) are presented in Table 1. There were large differences between the F0 lines (P < 0.001), the Leghorn and the Fayoumi lines appearing clearly as susceptible and resistant lines, respectively. All the Fayoumi birds survived from infection (*vs*. 26% mortality for the Leghorn) and showed on average one unit less severe lesions than the Leghorn chicks. The difference in WG was also important, being 3-fold higher in Fayoumi birds as compared to Leghorn birds. It should be clarified that the difference in WG was due to a difference in resistance to infection between the lines since non challenged (control) full brothers and sisters did not differ in WG (48.9% and 47.9% in control Fayoumi and Leghorn birds (P > 0.10), respectively, data not shown) in this study and previous ones with these lines [11].

**Table 1:** Elementary statistics on some resistance traits measured on inoculated animals (*E. tenella*) in the F0 (Leghorn and Fayoumi) and in their F1 cross, all F2 cross and selected susceptible (S) and resistant (R) F2

	Leghorn (n = 44)	Fayoumi (n = 44)	F1 (n = 104)	F2 (n = 860)	F2-S^1^ (n = 130)	F2-R^2^ (n = 130)
**Mortality (%)**^3^	26.7	0.0	0.0	1.4	4.6	0.0
**LES**^4^						
mean ± sd	3.7 ± 0.6	2.9 ± 0.6	2.9 ± 0.6	3.1 ± 0.6	3.4 ± 0.6	2.5 ± 0.8
min/max	2/4	2/4	1/4	1/4	2/4	1/4
**WG (%)**^5^						
mean ± sd	11.1 ± 13.3	32.6 ± 14.9	42.2 ± 15.2	32.0 ± 16.6	4.5 ± 8.0	50.6 ± 5.9
min/max	-21.7/49.2	2.6/61.9	-2.6/72.3	-14.5/68.2	-14.5/24.4	36.2/68.2

^1^F2-S = 15% selectively genotyped as the most susceptible within F2 families; ^2^F2-R = 15% selectively genotyped as the most resistant within F2 families; ^3^Mortality and ^4^LES = Lesions measured 8 d post inoculation (pi); ^5^Weight Gain (%) = 100 × (Body Weight (8 d post inoculation) - Body Weight (2 d before inoculation))/Body Weight (2 d before inoculation) (%)

It was relevant to compare the resistance of the F0 lines with the levels of the F1 and F2 cross although the tests were not performed at the same time but in identical conditions. For the resistance traits measured, the F1 cross was as resistant as the resistant Fayoumi, showing no mortality and a low depressing effect of the infection on the WG. The F2 cross showed resistance levels closer to the resistant Fayoumi or to the F1 cross than to the mid-range values between the two founder lines. The F2 cross displayed the largest range of LES (1 to 4) and of WG (-14.5% to 68.2%). As the strategy of selective genotyping was applied, values of the lower (F2-S) and upper (F2-R) groups are given. Selection was primarily applied on WG, which created two non overlapping groups with high differences in WG of 6.6 sd (P < 0.001). The F2-S and F2-R groups were slightly overlapping for LES values, differing by an average of 1 unit of lesion.

Phenotypic correlations between the resistance traits measured in the whole F2 and further included in the QTL analysis are presented in Table 2. Correlations were all in the expected direction but of various strengths. The strongest and most significant correlation was observed between the WG and the plasma coloration (PC) (0.70, P < 0.001). Also highly significant (P < 0.001) but more moderate values of correlations (from 0.39 to 0.56) were observed between WG and PC with LES and hematocrit level (HEMA), and between HEMA and LES. Rectal body temperature (T°) showed lower correlations with HEMA (0.14, P < 0.001) and with WG and PC (0.09, P < 0.01).

**Table 2:** Phenotypic correlations between resistance traits included in the QTL analysis measured on inoculated animals (*E. tenella*) in the whole F2 cross

	PC^1^	T°^2^	HEMA^3^	LES^4^
WG^5^	0.70**	0.09*	0.56**	-0.44**
PC^1^		0.09*	0.56**	-0.48**
T°^2^			0.14**	ns
HEMA^3^				-0.39**

n = 860

*P < 0.01; **P < 0.001; ^ns ^= P > 0.10

^1^PC = Plasma Coloration 4 d post inoculation (log_10_(Optical Density at 480 nm)); ^2^T° = Rectal Body Temperature (°C) 4 d post inoculation; ^3^HEMA = Hematocrit Level (%) 4 d post inoculation; ^4^LES = Lesion (from 0 "no lesion" to 4 "most severe lesion") 8 d post inoculation; ^5^WG = 100 × (Body Weight (8 d post inoculation) – Body Weight (2 d before inoculation))/Body Weight (2 d before inoculation) (%)

### QTL analysis of resistance traits to *E. tenella *in the cross

Table 3 shows the location and density of markers on the chicken chromosomes (GGA) used in the QTL analysis. Tables 4 and 5 summarize the results of the QTL analysis, showing the location of the significant QTL, the flanking markers of this location, the maximum F value obtained at the location, the genetic effects of the QTL, the reduction of the residual variance obtained by fitting this QTL, and the chromosome-wide and genome-wide significance levels associated with this QTL. Table 4 presents the results for models including additive and dominance effects and Table 5 presents the results of QTL for which a parent of origin effect was found as significant.

**Table 3:** Number of markers, map length [48], first and last markers for each chromosome (*GGA*)

*GGA*	Marker number	Map length (cM)	First marker	Last marker
1	37	532	ADL0160	MCW0108
2	21	397	MCW0205	LEI0104
3	10	315	MCW0261	MCW0037
4	13	240	ADL0143	ADL0265
5	9	166	LEI0082	ADL0298
6	7	74	LEI0192	LEI0093
7	5	165	LEI0064	ADL0169
8	5	76	MCW0275	LEI0136
9	4	84	LEI0028	MCW0134
10	7	58	MCW0194	LEI0103
11	3	37	LEI0072	ADL0308
12	3	90	ADL0372	MCW0332
13	3	48	MCW0213	ADL0214
14	4	72	MCW0296	LEI0066
15	6	47	MCW0226	MCW0323
17	3	32	ADL0293	ADL0199
18	3	40	ADL0304	MCW0219
23	3	10	MCW0165	LEI0090
24	3	28	ROS00302	LEI0069
26	4	41	ADL0330	LEI0074
27	2	26	MCW0233	MCW0328
28	2	22	ADL0341	MCW0227

**Table 4:** Estimation of QTL for resistance traits to coccidiosis after inoculation with E. tenella in an F2 cross.

QTL region^1^	Flanking markers	Trait^2^	Location (cM)	F	Additive effect ± se	Dominance effect ± se	Corrected Additive effect ± se	Corrected Dominance effect ± se	Reduction of σ^2 ^(%)	Chrom-Wide Proba	Genome-Wide Proba
**1-A**	SEQALL0480-SEQALL0478	WG	216	8.79	-8.34 ± 2.44	8.29 ± 4.09	-3.20 ± 0.93	3.18 ± 1.57	6.5	0.005	0.024**
		PC	216	6.88	-0.188 ± 0.056	0.109 ± 0.092			5.1	0.014	0.066*
		HEMA	215	11.44	-1.788 ± 0.528	ns			4.3	0.009	0.043**
**1-B**	MCW0101-LEI0101	WG	254	17.42	-9.57 ± 2.29	ns	-3.67 ± 0.88	ns	6.4	0.0003	0.001***
		PC	254	10.90	-0.173 ± 0.052	ns			4.1	0.0039	0.019**
**2-A**	MCW0173-ADL0267	T°	248	11.15	0.146 ± 0.044	ns			4.2	0.016	0.10*
**2-B**	ADL0236-MCW0185	WG	297	6.78	-7.41 ± 2.52	7.05 ± 3.92	-2.84 ± 0.97	2.70 ± 1.50	5.1	0.024	0.15^ns^
**3-A**	LEI0161-MCW0212	T°	143	8.21	-0.138 ± 0.048	ns			3.1	0.045	0.31^ns^
**3-C**	GCT053-MCW0040	WG	281	5.92	-7.42 ± 2.23	3.74 ± 3.55	-2.84 ± 0.85	1.43 ± 1.36	4.5	0.042	0.30^ns^
		PC	281	7.86	-0.143 ± 0.051				3.9	0.050	ns
**6-A**	MCW0250-SEQALL0472	HEMA	59	10.61	-1.622 ± 0.498	ns			4.0	0.0062	0.19^ns^
		PC	66	15.55	-0.207 ± 0.052	ns			5.7	0.0011	0.038**
**6-B**	SEQALL0472-SEQALL0486	WG	79	5.56	-7.78 ± 2.42	-4.88 ± 3.71	-2.98 ± 0.93	-1.87 ± 1.42	4.2	0.030	0.65^ns^
**15**	MCW0211-MCW0323	WG	50	6.7	6.56 ± 2.53	ns	-3.20 ± 0.93	ns	2.5	0.044	ns
		HEMA	50	9.54	1.728 ± 0.559	ns			3.6	0.010	ns
		LES	50	7.66	-0.248 ± 0.090	ns			2.9	0.025	ns
**23-A**	MCW0165-ADL0289	T°	2	3.94	0.109 ± 0.047	-0.128 ± 0.073			3.0	0.050	ns
**23-B**	ADL0289-LEI0090	WG	8	5.5	5.87 ± 2.50	ns	2.25 ± 0.96	ns	2.1	0.044	ns

^1^QTL region = Chromosome number and letter to define sub-region; ^2^WG = 100 × (Body Weight (8 d post inoculation) – Body Weight (2 d before inoculation))/Body Weight (2 d before inoculation) (%); PC = Plasma Coloration 4 d post inoculation (log_10_(Optical Density at 480 nm)); T° = Rectal Body Temperature (°C) 4 d post inoculation; HEMA = Hematocrit Level (%) 4 d post inoculation; LES = Lesion (from 0 "no lesion" to 4 "most severe lesion") 7 d post inoculation;

Genome-wide probabilities thresholds are indicated as very significant*** (P = 0.001), significant** (P < 0.05), suggestive* (P < 0.10) or non significant ^ns ^(P > 0.10).

QTL with additive and dominance effects.

**Table 5:** Estimation of QTL for resistance traits to coccidiosis after inoculation with *E. tenella *in an F2 cross.

QTL region^1^	Flanking markers	Trait^2^	Location (cM)	F	Additive effect ± se	Dominance effect ± se	Parent of Origin effect ± se	Reduction of σ^2 ^(%)	Chrom-Wide Proba	Genome-Wide Proba
**1-C**	ROS0313-MCW0283	WG	404	6.74	5.40 ± 2.22	-6.77 ± 3.66	-8.85 ± 2.47	7.4	0.0076	0.036**
		PC	402	7.41	0.099 ± 0.050	-0.174 ± 0.081	-0.220 ± 0.056	8.1	0.0040	0.019**
**3-B**	MCW0252-ADL0306	WG	220	5.46	-5.83 ± 2.79	1.84 ± 5.16	9.04 ± 2.78	6.1	0.018	0.14^ns^

^1^QTL region = Chromosome number and letter to define sub-region; ^2^: WG = 100 × (Body Weight (8 d post inoculation) – Body Weight (2 d before inoculation))/Body Weight (2 d before inoculation) (%); PC = Plasma Coloration 4 d post inoculation (log_10_(Optical Density at 480 nm)); inoculation;

Genome-wide probabilities thresholds are indicated as significant ** (P < 0.05) or non significant ^ns^(P > 0.10).

QTL with parent of origin effects.

The QTL analysis detected 21 chromosome-wide significant (P < 0.05) QTL on 13 QTL regions of 6 chromosomes. Out of these, 1 QTL was genome-wide very significant (P = 0.001), 6 QTL were genome-wide significant (P < 0.05) and 2 QTL were genome-wide suggestive (P < 0.10).

### QTL with dominance (and additive) effects

Genome-wide significant QTL were found mostly on the longest chromosomes: five on GGA1, one on GGA2 and one on GGA6 (see Table 4). Significant QTL for WG were obtained on all QTL locations found in this study, the most significant being on GGA1 at 254 cM (1-B region) with a genome-wide significance of P = 0.001. Another genome-wide significant QTL was found for WG at 216 cM (1-A region) and chromosome-wide significant QTL for WG were identified on GGA2, GGA3, GGA6, GGA15 and GGA23. A genome-wide suggestive QTL was found on GGA1 for PC at 216 cM (1-A region) as well as two genome-wide significant QTL at 254 cM (1-B region) and on GGA6 at 66 cM (6-A region); one chromosome-wide significant QTL for PC was identified on GGA3. A genome-wide significant QTL was found on GGA1 for HEMA at 215 cM (1-A region) and chromosome-wide significant QTL for HEMA were identified on GGA6 and GGA15. A genome-wide suggestive QTL was found for T° at 248 cM (2-A region) and chromosome-wide significant QTL for T° were identified on GGA3 and GGA23. There was one chromosome-wide significant QTL for LES obtained on GGA23.

In most of the cases and mainly for the most significant QTL having the largest effects, the additive effects were negative for WG, PC, HEMA and T°, indicating that the favourable alleles were coming from the Fayoumi resistant line. The exceptions were the genome-wide suggestive QTL for T° on GGA2 and all the five QTL identified on GGA15 and GGA23 of lower significance. Out of the 18 QTL described above, dominance was only significant for six of them and two were associated with suggestive or significant genome-wide effects only. In four cases, including the two most significant QTL mentioned previously, dominance was positive, indicating a superiority of the heterozygote over the midparent. The WG trait was present in four of the six cases.

The reduction of the residual variance when fitting the QTL in the models varied from 2.1% to 6.5%, the highest value being associated with the two QTL for WG on GGA1 and the QTL for PC on GGA6.

### QTL with "imprinting" effects

"Imprinting" or "parent of origin effect" was found to be very significant for WG and PC on GGA1 around 403 cM (1-C region) and to a lesser extent for WG on GGA3 at 220 cM (3-B region). On GGA1, the QTL was genome-wide significant (P < 0.05) and for the 3 QTL, the reduction of the residual variance when fitting the QTL in the models was higher than for the additive and dominance genetic effects described previously, varying from 6.1% to 8.1%. For the QTL on WG and PC on GGA1, the additive effects were positive but dominance effects and parent-of-origin effects were negative whereas for the QTL for WG on GGA3, the effects were all in the opposite direction.

### Chromosome GGA1

**Figure 1:**
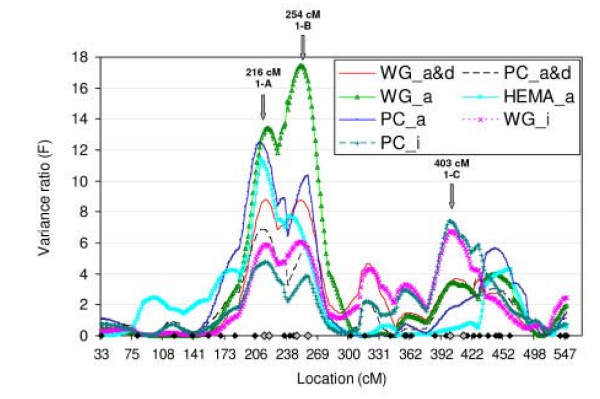
**Interval mapping of QTL on chromosome *GGA1***. Only the three traits with QTL detected on *GGA1 *are shown: WG (Body Weight Gain), PC (Plasma Coloration) and HEMA (Hematocrit level). Variance ratios (F) are shown for the different models used to identify the QTL: a&d (additive and dominance effects), a (additive effect) and i (parent of origin effect). For example, BW_a&d represents the Body Weight Gain trait using an additive and dominance effect model. Positions of the markers are indicated by black lozenges and flanking markers of the QTL identified (pointed by an arrow) are indicated by grey lozenges. Corresponding significance levels of the QTL are given in Table 4.

Since the three models (additive, additive and dominant, imprinting) were all used on GGA1 for identifying QTL for BW and PC, GGA1 was chosen to illustrate and compare F distributions. Figure 1 shows the F distribution for the traits and the models for which QTL have been found (see corresponding results on Tables 4 and 5): HEMA with additive (a) model, and WG and PC with additive ("a"), additive and dominant ("a&d") and imprinting ("i"). Three locations were identified with each two QTL for WG and PC: 216 cM (1-A region) with "a&d" QTL, 254 cM (1-B region) with "a" QTL and 402 cM (1-C region) with "i" QTL (Tables 4 and 5). On the 1-A and 1-B regions, distributions for "a" model were always superior to the "a&d" and "i" models but indicated the same locations for the putative QTL. For the 1-C region bearing the "i" QTL, maximum F values corresponded to the same QTL location for WG only.

## Discussion

Chicken lines like the Fayoumi and Leghorn lines, showing large differences in resistance to *E. tenella*, represent unique resources for searching for genes controlling disease related traits and underlying mechanisms. From these lines, an F2 resource population was produced, challenged and genotyped and several QTL were found for different disease resistance traits which will be discussed first.

### Resistance traits for *E. tenella*

The resistant Fayoumi line and the susceptible Leghorn lines differed significantly for a number of resistant traits. The same traits were informative in the F2 cross and could possibly be combines to an index to assess resistance to *E. tenella*. The highest correlation was observed between WG and PC and it can be questioned whether PC would be a good indicator to individually follow the kinetics of resistance to *E. tenella *or assess differences between genetic groups since it is an easy measure. Plasma coloration measures the effect of the parasitic infection on the loss of carotenoid pigments and may be more appropriate from a physiological point of view as an indicator for intestinal infections like *E. maxima *than caecal ones like *E. tenella *[17]. Within the pure F0 lines, correlations between WG and PC showed a different level according to the resistance of the line, being higher within the resistant Fayoumi line than in the susceptible Leghorn line [11]. In studies with *E. maxima*, correlations also seemed to vary with the inoculation dose [17,18]. In practice, it seems difficult to use a unique disease phenotype or index of phenotypes but possible to adjust it case by case on a combination of level of resistance of the host and severity of the infection.

### Method

In the line cross method used here, founder lines are assumed to be fixed for alternative alleles and QTL found explain the genetic variation between the F0 lines [19]. We were not strictly in this situation, though the Fayoumi originating from Egypt and the White Leghorn lines are distant and quite different breeds as shown by the high level of heterozygosity (above 76%) of the microsatellite markers tested in the F1 fathers (data not shown).

The QTL analysis on chromosome GGA1 clearly showed the importance of the model chosen to analyze the data as for the three locations and traits, either a model with dominance effect, or additive and dominance effects, or including a parent-of-origin effect fitted the data the best. Using an incorrect model would lead to different results, as illustrated here (see Figure 1), due to different level of significance and even different locations. The example of GGA1 also shows the limitations of the models since methodological difficulties are arising with several QTL on the same linkage group fitting different models and possibly in interaction. More complex methods not available yet on QTL Express will be needed to perform such multi-QTL analyses.

Selective genotyping can be an efficient method, providing good power as compared to complete genotyping at a reduced cost [20]. This method has been widely used, with sometimes adjustments from the original strategy, in QTL mapping half-sibs or F2 designs, for different species and different traits, such as growth or immune related traits [e.g. [21-24]]. It may be argued that using regression interval mapping (and not maximum likelihood analysis) on selectively genotyped animals might lead to an estimated bias [25]. In fact, the regression method that we used does not bias the estimation of the location of the QTL and there is no risk of detecting spurious QTL [26]. The only risk is to overestimate the parameters as shown in real data [21]. To compensate for this likely overestimation of the QTL effect, a correction factor was applied [20] on WG and, in theory, should also be applied to the correlated traits [27]. Only, we did not apply strict truncation selection within the whole F2 but within each F2 family and this theoretical correction factor is likely too stringent. Indeed, the reason and advantage of selecting extreme individuals within families was to exploit the quantitative variation which is in linkage disequilibrium with the markers coming from the within-family genotypic variation [28], allowing at the same time a better coverage of the whole F2 population.

### QTL effects

All QTL locations found in this study included at least a QTL for WG. This could be expected since F2 animals were selectively genotyped to give most power to detect QTL for this trait. Also, this trait showed larger variation as compared to the T° for instance, for which two QTL were identified only. In case of LES, the lack of an appropriate model taking into account the variable as discrete may have hampered the QTL detection. Still, genome-wide suggestive or significant QTL were found for WG, PC, HEMA and T°, i.e. all the traits except LES for which only chromosome-wide significant QTL were identified.

The additive effects found in this study estimated differences between the Fayoumi and the Leghorn lines. In most cases, the additive effects were negative indicating that the favourable alleles were coming from the Fayoumi resistant line, as one could expect. Also, in the most significant cases, dominance was positive, indicating a superiority of the heterozygote over the midparent. This result could be logically related to the observation of the F1 being as resistant as the resistant Fayoumi line. The two genome-wide significant QTL and to a lesser extent the two chromosome-wide significant QTL which were identified with dominance effects could be used in practice to maximize crossbreeding performances by using QTL with dominance variation [29].

The lines being reciprocally crossed allowed testing for parent of origin effects in the F2. Indeed, QTL for WG and PC with significant parent-of-origin effects were found on GGA1 (around 403 cM) and for WG on GGA3 (around 220 cM). This parent-of-origin effect is intriguing in the chicken since it can only resemble genomic imprinting observed in mammals but has been evidenced in a few studies concerning egg or body weight traits [30] and interestingly, meat quality characteristics for which the QTL was identified in the same region of the GGA3 (around 225 cM) [31]. Parent-of-origin expression may explain reciprocal effects when found. In our case, clear differences between F1 reciprocal crosses were observed, not for LES, but for PC, feed conversion and especially for WG, both F1 males and F1 females originating from a Fayoumi mother being more resistant than contemporary birds coming from a Leghorn mother, showing 20% and 30% more WG, respectively (unpublished data). This difference between reciprocal crosses had been attributed to maternal effects. Here, both QTL on GGA1 and GGA3 might not explain all this observed difference and, moreover, shown effects of opposite directions. In most cases, the reciprocal effects showed are likely a combination of both maternal effects and parent of origin effects [32]. There is growing evidence of the importance of parent of origin effect as a source of genetic variation in other species, like in sheep [33] and more examples are likely to come for disease resistance related traits. In our case, further investigation is needed to confirm this parent of origin effect with more appropriate models, than the one used here, assuming founder lines fixed for alternative alleles [33].

### Co-localization of QTL

On the three QTL locations of GGA1, on GGA3, GGA6, GGA15 and GGA23, several QTL for different resistance phenotypes were identified on the same location or very close by. This situation occurred for two or three traits, not always the same but corresponding to the highest F value for other traits also, although not reaching significant levels (data not shown). This co-localisation of QTL is not surprising since all these traits have been shown to be correlated. It seems a reasonable assumption that different mechanisms lead to differential expression of the disease, this is reminiscent of the involvement of some common paths and thus common genes. These common pathways and genes are not only highly interesting to be identified from a biological point of view but could also be very promising to be used in practice to simultaneously improve several disease resistance traits.

### Comparison with other QTL analysis

In the last decades, there has been an accumulating number of QTL mapping studies in the chicken, using mostly F2 designs [reviewed by [34,35]] and applied mainly on production traits like growth related traits. Fewer concern disease related traits. Even in these cases, it is difficult and even hazardous to try to compare results using different traits and often finding large intervals. Interestingly, in one review [35], an attempt is made to use ontology terms allowing to search for "Disease Resistance" related QTL. Comparable QTL regions with the ones we identified in the present study were shown for primary response to SRBC on GGA1 but in a rather large marker bracket including our 1-C region [36,37] and again for antibody response in two different studies on GGA6 [36-38]. In addition, a suggestive QTL for viremia to Marek's disease was found in the same 1-B region of GGA1 close to LEI0101 [39].

The only really pertinent comparison which can be performed is with, to our knowledge, the only other genome scan for QTL for resistance to coccidiosis [15], to another *Eimeria *species, *E. maxima*. Using 314 F2 birds and 119 microsatellite markers on 16 linkage groups (including 9 chromosomes), one significant QTL was identified on oocyst production, exactly around the same location, 252 cM, near LEI0101, corresponding to the chromosome-wide significant additive QTL we found on GGA1 at 254 cM (1-B region: MCW0101 – LEI0101). This QTL on oocyst production was confirmed by an additional fine-mapping study, enriched by 8 additional microsatellite markers [16]. In this study and ours, the exact position remains to be identified but it is very interesting to consider the possibility of having a unique QTL for resistance to two different *E*. species.

### Possible candidate genes

From this QTL study, only intervals from 5 to 25 cM were identified. So, it is, of course, not possible to already identify putative candidate genes. Still, it is interesting to look at the genomic regions identified using genome browsers as Map Viewer (National Center for Biotechnology Information, ) or UCSC Genome Browser . Descriptions, interactions and functions of candidate genes have been explored through the use of Ingenuity Pathway Analysis (Ingenuity^® ^Systems, ) and OMIM database (Online Mendelian Inheritance in Man, John Hopkins University and National Center for Biotechnology Information, ). A list of possible candidate genes in QTL intervals is presented in Table 6. The first candidate genes we would look at might be innate immunity and inflammatory genes playing a role during initial pathogen exposure as birds were challenged for primary infection. It is noteworthy that right in the 1-B region (at 254 cM) a QTL for oocyst production to *E. maxima *was reported [15,16]. The TNF receptor superfamily member 1A (TNFRSF1A) is located there and could be related to the role of TNF in inflammatory and immune response to the parasite. The level of TNF during *Eimeria *infection displays two peaks, and it is considered that the first one is related to inflammation during the onset of the disease, and that the second one is related to immunity setup [40]. In the 1-C region corresponding to the parent-of-origin QTL lies an interesting innate immune gene from the TLR pathway, the toll-like receptor 7 (TLR7). Interleukin 17 receptor A (IL17RA) is located in QTL interval 1-A, and it is particularly interesting to note that pro-inflammatory cytokine IL17D is over-expressed notably in gut and spleen during *Eimeria maxima *infection in chicken [41].

**Table 6:** Putative candidate genes in QTL regions

QTL region	candidate gene name	description	functional category	example of activity
**1-A**	BCL2L13	BCL2-like 13 (apoptosis facilitator)	apoptosis	regulated by TNFα, pro-inflammatory cytokine
	IL17RA	interleukin 17 receptor A	inflammatory response	receptor of IL17, pro-inflammatory cytokine
**1-B**	TNFRSF1A	tumor necrosis factor receptor superfamily, member 1A	inflammatory response	receptor of TNFα, pro-inflammatory cytokine
**1-C**	TLR7	toll-like receptor 7	inflammatory response, innate immunity	recognizes pathogen-associated molecular patterns
**2-A**	GRB10	growth factor receptor-bound protein 10	growth	regulates IGF-1 signaling
	IKZF1	IKAROS family zinc finger 1 (Ikaros)	immune response	triggers CD4/CD8 commitment lineage
**2-B**	ANKRD12	ankyrin repeat domain 12		regulated by CXCL12
**3-B**	GHRL	ghrelin/obestatin preprohormone	growth	regulates GH
**3-C**	APOB	apolipoprotein B (including Ag(x) antigen)	lipid transport	principal component of LDL
**6-A**	CXCL12	chemokine (C-X-C motif) ligand 12 (stromal cell-derived factor 1)	inflammatory response, immune response	activates leukocytes
**6-B**	BLNK	B-cell linker	immune response	role in B-cell function and development
**23-A**	FABP3	fatty acid binding protein 3, muscle and heart (mammary-derived growth inhibitor)	lipid transport	participates in uptake and transport of fatty acids
	C2	complement component 2	innate immune response	part of complement system pathway

**Figure 2:**
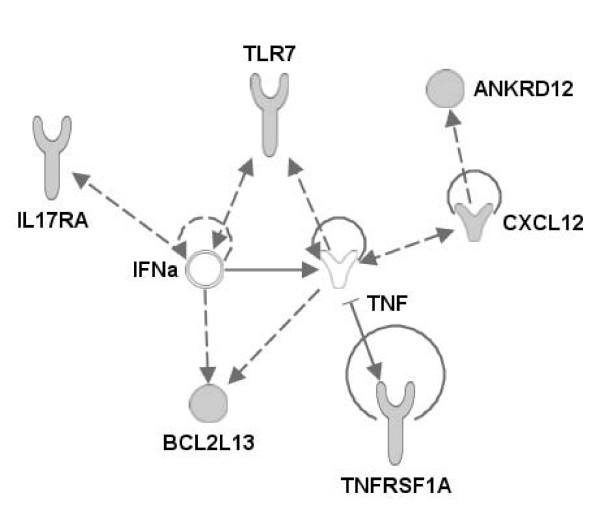
**Network of interaction between products of candidate genes**. The network illustrates molecular interactions between products of candidate genes selected from QTL regions. Arrows with plain lines represent direct interactions, and arrows with interrupted lines represent indirect interactions. Relations have been determined using information contained in the Ingenuity Pathways Knowledge Base.

Using the list of candidate genes, it is possible to derive a network of molecular interactions between gene products to illustrate the functional pathways involved in response to *Eimeria *that could be in the QTL regions. The network has been generated through the use of Ingenuity Pathways Analysis (Ingenuity^® ^Systems, ) and is represented in Figure 2. The network illustrates that several candidate genes are involved in innate immune and inflammatory response through IFN and TNF nodes. It has been demonstrated that the levels of expression of many cytokines, including TNFα, TNFγ, IL-6, IL-17 increase dramatically in intestinal lymphocytes during primary infection to *E. tenella *or *E. acervulina *[42]. We can then conclude that the candidate genes from the QTL regions are relevant regarding response to *Eimeria *infection, and will need further investigation.

Lipid transport and metabolism should be investigated too, as two candidate genes from QTL regions are important for this pathway: FABP3 (23-A region) and APOB (3-C region). Apolipoprotein-B (APOB) is located in a QTL interval controlling for phenotypes T°, WG and PC. APOB is the main apolipoprotein constituting LDL and VLDL for lipid transport, and then plays an important role in fat absorption from diet and energetic metabolism. It has been demonstrated that polymorphisms of APOB gene are associated in broiler lines with weight gain and obesity [43]. Moreover, carotenoids are distributed to tissues through lipoprotein transport, and the plasma coloration (PC), measured by absorbance at 480 nm reflects the level of carotenoid. Then this trait can be influenced by variation of APOB. Recently, genetic polymorphisms of APOB have been associated to variation in carotenoid plasma level in human [44]. Genetic variation at APOB locus can then explain the variability of two correlated traits, PC and WG, by differential absorption and distribution of lipids, including carotenoids.

Again, these are only observations in the identified QTL regions and further work is needed to finer map these QTL but in some cases it could bring together structural evidence with accumulating knowledge on the implication of chemokines, cytokines and other proteins in response mechanisms to *Eimeria *infections.

## Conclusion

From an F2 cross from resistant and susceptible chicken lines, the QTL analysis detected 21 chromosome-wide significant QTL on 8 chromosomes. Out of these, 9 were genome-wide very significant to suggestive. Candidate genes and relevant pathways were suggested. This study is a good starting point for further gene identification and delineation of underlying mechanisms and hopefully opening possibilities for new breeding strategies including improved resistance to coccidiosis in the chicken. Additional analyses are now initiated to investigate the QTL regions identified so far. The next steps will be done in parallel: marker density will be dramatically increased by using high density SNP panels available now and on the whole F2. Analysis models will be improved to assess the potential pleiotropic nature of QTL on the different traits by building multitrait and multi-QTL models. A complementary approach will explore functional genomics [45] by utilizing variation of gene expression between the Fayoumi and Leghorn lines. *In fine*, both structural approaches as illustrated in this study and coming functional data will be combined to provide positional candidate genes for resistance loci for coccidiosis.

## Methods

### Origin of the F0 lines and F1 and F2 populations

The strategy chosen to identify markers of resistance to coccidiosis was to perform an F2 cross originating from F0 lines showing extreme phenotypes and to search for markers of resistance in this cross. After the screening of several lines for their resistance to *E. tenella*, a resistant line (Fayoumi) and a susceptible line (Leghorn) were identified [11]. An F1 cross was produced using 3 cocks and 7 hens from the Fayoumi line and 3 cocks and 6 hens from the Leghorn line. From this F1, 104 animals (males and females) were tested for *E. tenella*. Non-challenged full brothers and sisters were used to produce the F2 cross: 6 F1 cocks, each mated to 5 F1 hens, being sisters or half-sisters, produced 860 F2 (males and females).

### Challenge and Resistant traits recorded

The results presented here for the F0, F1 and F2 animals correspond to a test for resistance to *E. tenella *at the AFSSA Parasitology Unit of Ploufragan following strictly the same challenge protocol. Chickens were weighed at 26 d and separated in cages, within lines (for F0), sex and dam family, by groups of four chicks of similar body weight. Birds were inoculated *per os *at 28 d of age with a dose of 50.000 oocysts of *Eimeria tenella *from the PT_5 _strain (maintained at the Parasitology Unit of Ploufragan since 1965) since this dose had been shown to display the largest difference in resistance between the two F0 lines [46]. Challenged birds were slaughtered and weighed 8 d after inoculation at 36 d of age. The following resistance criteria were measured: Mortality was recorded until 8 d after inoculation. Body weight gain (WG) was measured as WG = 100 × (Body Weight (8 d post inoculation) – Body Weight (2 d before inoculation))/Body Weight (2 d before inoculation). From blood sampling at 4 d post inoculation, plasma coloration (PC), as a measure of blood carotenoid level, was analyzed as PC = log_10_(Optical Density at 480 nm) [17] and hematocrit level (HEMA %) was recorded. Rectal body temperature (T°) was measured 4 d post inoculation. At slaughter, cecal lesion scores (LES) were assessed from 0 (no lesion) to 4 (most severe lesions) [47].

### Genotyping

Genomic DNA was extracted from red cells by phenol chloroform extraction. Genotyping was performed in two steps. The 6 F1 fathers were first tested with more than 300 microsatellite markers in order to identify markers, which were informative in the cross, using an automatic sequencer ABI 377 (Perkin-Elmer). Markers were chosen to obtain the best genome coverage [48]. An initial set of 139 markers was chosen and organized in 24 sets of markers to perform the multiplex typing Profiles were then analyzed and validated in genotypes using the Genotyper^® ^Analysis 3.7 software (Applied Biosystems, Foster City, CA USA). The GEMMA database was used to manage the informativity tests [49]. The strategy of "selective genotyping" [20] was applied by individual typing of extremes. Within each F1 mother families (and thus F1 father also), the 15% most resistant and the 15% most susceptible were genotyped. Each F1 mother had about 28 F2 offspring tested, the 4 most resistant and the 4 most susceptible being typed, sex ratio being balanced in all cases. To choose the extremes, the first chosen criterion was WG, then LES. All F0 (n = 19) and F1 (n = 36) animals used to produce the cross and selected F2 animals (n = 260) were genotyped for all markers. After this genome scan, QTL regions were identified and 18 new informative markers out of 38 tested were added in 7 putative QTL regions. These markers were already available or selected for their informativity among microsatellites developed for this study from the chicken genome assembly (, see Table 7). The results presented here are the outcome of the complete analysis using a total of 157 microsatellite markers covering 22 chromosomes (see Table 3).

**Table 7:** New microsatellite markers developed from the chicken genome assembly (galGal3, ).

Marker name	Position (galGal3 assembly)	Upper primer	Lower primer	PCR annealing T°
SEQALL0348	chr1:135392697-135392876	GCTCAGCACCTCCTCCTC	AGAAAGCAGCCTCACAAAGC	55°C
SEQALL0349	chr1:135543677-135543882	AGGGTTTCCAAGTGGTGTTG	ACCTTGCCTGAGACTGGTG	55°C
SEQALL0350	chr1:135576325-135576564	CGACAGATGGTCAAGAATGG	ACACAGTTCTTCGCTGTACG	55°C
SEQALL0468	chr1:64162253-64162451	TTGCCATTCGAAACATCAAC	GGACTCTGCTGTGCCAAATAC	50°C
SEQALL0469	chr1:64480773-64480997	TGAGATGATGAATGGCTTGG	ATATGCAGCAGGGCTCATTC	58°C
SEQALL0470	chr1:72300927-72301126	CACAGGTCCACGAGAAAAGG	TGCAGTCCTTCACATTCTGC	58°C
SEQALL0471	chr1:78494600-78494914	AGCATCATGACAGTCCAACC	AGGCCAAATTGTCTCACTCG	58°C
SEQALL0472	chr6:24021693-24022070	ATCATGCGGCAGAAAAAGAG	AAATCCATCAGCCTGACAGC	50°C
SEQALL0473	chr6:24772426-24772628	GAGAGGGAGGGAGATGAAGG	GAAGAGCTCGTGCAAAAAGC	58°C
SEQALL0474	chr6:25154425-25154640	TAGGGTTTGGCTGTTGTCTG	CATTTGGAAACCCAGAGCAG	58°C
SEQALL0475	chr15:4463535-4463868	ACAGGATGCTGCTGCTCAC	AATGTTTCCCTTTCCCAACC	58°C
SEQALL0476	chr15:8651118-8651325	AGCCAAATCCTGTTTCATGC	TGTCCTTTCTGGGAGAGACG	58°C
SEQALL0477	chr15:9172235-9172553	CATCCAAGTAACCCCACCTG	TGTAAGGTGTAGGGCGTTGG	58°C
SEQALL0478	chr1:72181285-72181516	TGGCTTATGGCAACAAAAATC	TGGCCGGTAGTGGATAAAAG	58°C
SEQALL0479	chr1:68536797-68537124	GATGTTCCTGGCACTTATAGGG	TTTCCCTTTGTTTCTGCATCC	58°C
SEQALL0481	chr1:142664771-142665102	GCAGCTGCTGTTCCACAAG	GCTCATTGCATTTTTGCTTTC	58°C
SEQALL0482	chr2:88572048-88572376	GGAAGTGTGTGTTATGGACTGG	CTAGGCTGGAACTGGCAGTAG	58°C
SEQALL0483	chr2:90911596-90911826	TCTTGGAGATCACGGGAAAG	TGCAACATCAGGACAAGAGG	58°C
SEQALL0484	chr6:5752041-5752395	CGAGTCTGCTGAATGGACAC	GCGCAATTAATTCAGGAAGG	58°C
SEQALL0485	chr6:6245960-6246304	GACCACACAGTCACACGTCAG	TCTGGAGGAAGCAATAAGAAGTG	58°C
SEQALL0486	chr6:25680338-25680656	TTCCCCAGGTGCTCTATCAG	ACCATCGTCTCCTGCCATAG	58°C

### QTL analyses

QTL analyses were performed using the line cross method from QTL Express software [19]. For each trait on each chromosome analyzed, QTL were searched applying a regression analysis. First a model including an additive effect and a dominance effect was tested. If the dominance effect was not significant, it was removed in a second step. The third step checked for the parent of origin effect. In the models used here, a positive value for the additive effect indicated that the increasing allele originated from the Leghorn line. A positive value for the dominance effect indicated that the heterozygote was larger than the midparent. All models included a hatch effect (3 classes) and a sex effect as fixed effects. The maximum F value indicated the most likely location of the putative QTL.

Since selective genotyping was applied, parameter estimates obtained from regression may be upper biased and can be corrected by dividing by the following factor as suggested by Darvasi and Soller [20]: (1 + x × i), where x is the deviation of the truncation point from the mean and i is the mean of the selectively genotyped group of the truncated normal distribution (in sd units). For a selection of the 15% more resistant and 15% more susceptible, the dividing correction factor would be here (1 + 1.036 × 1.554) ≈ 2.610. Only in the present study, selection by truncation on WG was not performed strictly on the whole F2 population as in the method of Darvasi and Soller [20] but was applied within each F1 dam family so that all the families were represented in the QTL analysis. In the results, true values obtained from the regression analysis are given for all traits and in addition for WG, values corrected with the factor of 2.610.

The chromosome-wide significance levels (p_c_) were obtained carrying out 10000 permutations [50]. Only QTL identified with a p_c _< 0.05 were considered. The genome-wide significances (p_g_) were derived from chromosome-wide significance levels (p_c_), using an approximate Bonferroni correction: p_g _= 1 - (1 - p_c_)^1/r ^in which r was obtained by dividing the length of a specific chromosome by the length of the genome considered for QTL detection (2600 cM). Genome-wide very significant, significant and suggestive thresholds were set up at p_g _= 0.01, 0.05 and 0.10, respectively.

QTL analysis was performed on all the resistant traits recorded (ie, WG, PC, T°, HEMA and LES) except on mortality because of the too low level of mortality in the F2 (1.4%).

## Authors' contributions

MHP designed the study, wrote the paper, collected samples and performed the statistical analysis. BB supervised the molecular and functional aspects of the study, participated in the interpretation of the results and commented on an earlier draft. FP designed the first set of microsatellites and commented on an earlier draft. JLC, HL and KF carried out the genotyping analysis and interpretation. SL developed the new markers and performed their validation, AT carried out the genotyping analysis. DG supervised animal husbandry. JMR supervised coccidiosis challenges, organised and carried out the sample collection and measurements and commented on earlier drafts of the paper. PR supervised challenge protocols and commented on earlier drafts of the paper. All authors read and approved the final manuscript.
